# Endolysosomal Degradation of Allergenic Ole e 1-Like Proteins: Analysis of Proteolytic Cleavage Sites Revealing T Cell Epitope-Containing Peptides

**DOI:** 10.3390/ijms18081780

**Published:** 2017-08-16

**Authors:** Sabrina Wildner, Brigitta Elsässer, Teresa Stemeseder, Peter Briza, Wai Tuck Soh, Mayte Villalba, Jonas Lidholm, Hans Brandstetter, Gabriele Gadermaier

**Affiliations:** 1 Department of Molecular Biology, University of Salzburg, Salzburg 5020, Austria; sabrina.wildner@sbg.ac.at (S.W.); brigitta.elsaesser@sbg.ac.at (B.E.); teresa.stemeseder@sbg.ac.at (T.S.); peter.briza@sbg.ac.at (P.B.); waituck.soh@sbg.ac.at (W.T.S.); johann.brandstetter@sbg.ac.at (H.B.); 2 Christian Doppler Laboratory for Biosimilar Characterization, University of Salzburg, Salzburg 5020, Austria; 3 Departamento Bioquímica y Biología Molecular I, Universidad Complutense, 28040 Madrid, Spain; mayte@bbm1.ucm.es; 4 Thermo Fisher Scientific, Uppsala 75450, Sweden; jonas.lidholm@thermofisher.com

**Keywords:** Ole e 1-like proteins, endolysosomal degradation, homology modeling, docking experiments, cathepsin S, T cell epitope

## Abstract

Knowledge of the susceptibility of proteins to endolysosomal proteases provides valuable information on immunogenicity. Though Ole e 1-like proteins are considered relevant allergens, little is known about their immunogenic properties and T cell epitopes. Thus, six representative molecules, i.e., Ole e 1, Fra e 1, Sal k 5, Che a 1, Phl p 11 and Pla l 1, were investigated. Endolysosomal degradation and peptide generation were simulated using microsomal fractions of JAWS II dendritic cells. Kinetics and peptide patterns were evaluated by gel electrophoresis and mass spectrometry. In silico MHC (major histocompatibility complex) class II binding prediction was performed with ProPred. Cleavage sites were assigned to the primary and secondary structure, and in silico docking experiments between the protease cathepsin S and Ole e 1 were performed. Different kinetics during endolysosomal degradation were observed while similar peptide profiles especially at the C-termini were detected. Typically, the identified peptide clusters comprised the previously-reported T cell epitopes of Ole e 1, consistent with an in silico analysis of the T cell epitopes. The results emphasize the importance of the fold on allergen processing, as also reflected by conserved cleavage sites located within the large flexible loop. In silico docking and mass spectrometry results suggest that one of the first Ole e 1 cleavages might occur at positions 107–108. Our results provided kinetic and structural information on endolysosomal processing of Ole e 1-like proteins.

## 1. Introduction

Ole e 1-like proteins are relevant elicitors of type I allergy. To date, 15 allergens belonging to this protein family are officially acknowledged by the WHO/IUIS Allergen Nomenclature Sub-Committee and are listed in the AllFam database [1]. They are exclusively found in pollen, and hallmarks of this protein family are three conserved disulfide bonds and the [EQT]-G-x-V-Y-C-D-[TNP]-C-R consensus pattern. Recently, the first three-dimensional structure of an Ole e 1-like protein, Pla l 1 from plantain pollen, was solved, indicating a seven-stranded β-barrel stabilized by three disulfide bonds [2]. Natural Ole e 1-like proteins isolated from pollen exhibit partial *N*-glycosylations [3,4,5,6]. The biological function is to date unknown, but was proposed to be involved in fertilization processes based on homology and localization of LAT52, a cysteine-rich extracellular protein from tomato [7]. Before, a trypsin inhibitor function was suggested [8,9], but recent experimental data clearly ruled out such a function for Pla l 1. Instead, a role in cell wall modulation was proposed based on structural homology to proteins known to be involved in such processes [2].

The prototypic member of this protein family is Ole e 1, the major allergen of olive pollen, showing a sensitization prevalence of 80% among olive allergic patients [10]. Ole e 1 has been extensively characterized and both the protein sequence and attached oligosaccharide structures showed a high degree of polymorphism, which was suggested to contribute to its allergenicity [7,11]. Fra e 1 from ash pollen also constitutes the major allergen of the source and contributes mainly to spring pollinosis [5]. Ole e 1-like proteins exhibit a varying degree of sequence identity, which is typically high (>82%) among Oleaceae members, and hence, sensitization to Ole e 1 is used as a surrogate marker in the clinic [5,12]. In contrast, sequence identity to Ole e 1-like proteins from botanically-distant species is lower, ranging from 25–60%. Sal k 5 and Che a 1 are members from Russian thistle and chenopod, which both cause summer pollinosis predominately in dry and salt-rich habitats [13]. They represent important allergens with a sensitization prevalence of 30–40% and 77% in sensitized patients, respectively. A high degree of IgE cross-reactivity was observed between the two molecules while cross-inhibition with Ole e 1 was very limited [13,14,15]. Phl p 11 from timothy grass accounts for up to 32% sensitization among grass pollen allergic patients and constitutes only a minor allergen devoid of IgE cross-reactivity with Ole e 1 [2,8]. Pla l 1 is the major allergen of English plantain and typically demonstrates sequence identity lower than 40% with other Ole e 1-like allergens. Antibody cross-reactivity with members of other botanical families was shown to be absent or very limited [2,9,16].

T cell epitope mapping of Ole e 1 revealed residues 91–102, 109–120 and 119–130 as immunodominant T cell epitopes [17]. Notably, none of the other protein family members have been investigated regarding immunogenicity or T cell epitopes. An alternative to T cell epitope screening using synthetic peptides is the endolysosomal degradation assay. It has been previously shown that susceptibility to endolysosomal proteolysis by antigen-presenting cells could serve as a marker for protein immunogenicity [18,19,20,21,22]. Among others, Egger et al., used an in vitro assay to study immunogenic properties of proteins using endolysosomal proteases isolated from dendritic cells (DCs). Analysis of the endolysosomal fractions revealed that relevant endo- and exo-peptidases of the murine DC cell line JAWS II were comparable to those identified in human DCs. The most abundant proteases of the endolysosomal fraction are cathepsins, e.g., cathepsin B, D, E, L and S. While several cathepsins and other proteases are considered important for antigen processing and presentation, cathepsin S represents the major protease involved in this biological process [23,24].

The aim of this study was to investigate relevant allergens of the Ole e 1-like family regarding their susceptibility to endolysosomal degradation. Peptide clusters were determined by mass spectrometry, and corresponding cleavage sites were assigned to the primary and secondary protein structures. The relevance of cathepsin S during endolysosomal degradation was analyzed by specific protease inhibition. Computational docking between cathepsin S and Ole e 1 was used to investigate the cleavage behavior, potential binding sites and interaction properties in more detail.

## 2. Results

### 2.1. Ole e 1-Like Allergens Showed Different Susceptibility to Endolysosomal Degradation

**Figure 1:**
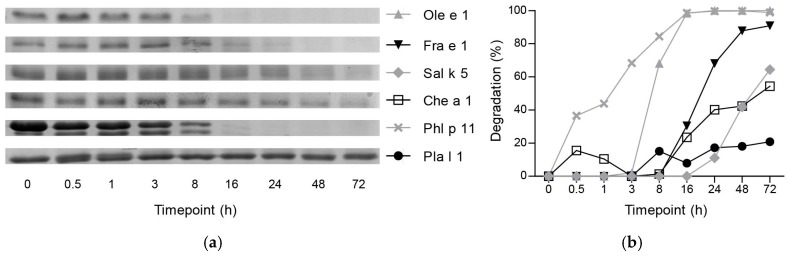
Degradation of intact protein upon endolysosomal degradation. (**a**) Gel electrophoresis of Ole e 1-like proteins upon incubation with endolysosomal proteases; (**b**) Percentage of degradation determined as intensity of protein bands in gel electrophoresis.

In order to investigate susceptibility to endolysosomal proteolysis, six representative allergens of the Ole e 1-like family (Table 1) were chosen. They belong to trees of the Oleaceae family (Ole e 1 and Fra e 1), weeds of the Amaranthaceae family (Sal k 5 and Che a 1), grasses (Phl p 11) and weeds of the Plantaginaceae family (Pla l 1). All allergens were recombinantly produced in *P. pastoris* and *Escherichia coli* and analyzed in vitro regarding their proteolytic stability using microsomal proteases isolated from murine dendritic cells. In gel electrophoresis, very high resistance was observed for Pla l 1, which was not significantly degraded even after 72 h of incubation. Sal k 5 and Che a 1 showed high stability with around half of the protein remaining intact after 72 h. Moderate susceptibility was observed for Ole e 1 and Fra e 1 with considerable degradation after 8 h. The fastest degradation was noted for Phl p 11 with significant proteolysis already after 0.5 h (Figure 1). Proteins originating from the same botanical families (e.g., Ole e 1/Fra e 1 and Sal k 5/Che a 1) generally showed similar degradation kinetics.

**Figure 2:**
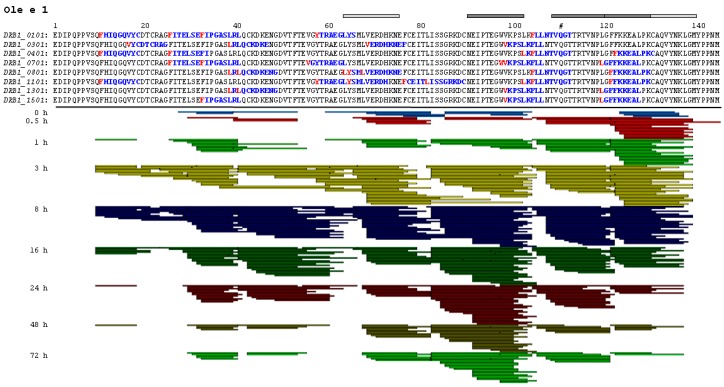
Endolysosomal degradation pattern and T cell epitopes of Ole e 1. T cell epitopes of eight *HLA-DRB1* alleles were predicted using the ProPred software and are shown in blue; the cleavage sites are highlighted in red. Peptide patterns obtained upon endolysosomal degradation are depicted from 0–72 h of incubation. Bars on top indicate dominant (dark grey) and minor (light grey) T cell epitopes identified by peptide mapping. #, glycosylation site lacking glycans due to amino acid exchange.

**Figure A1:**
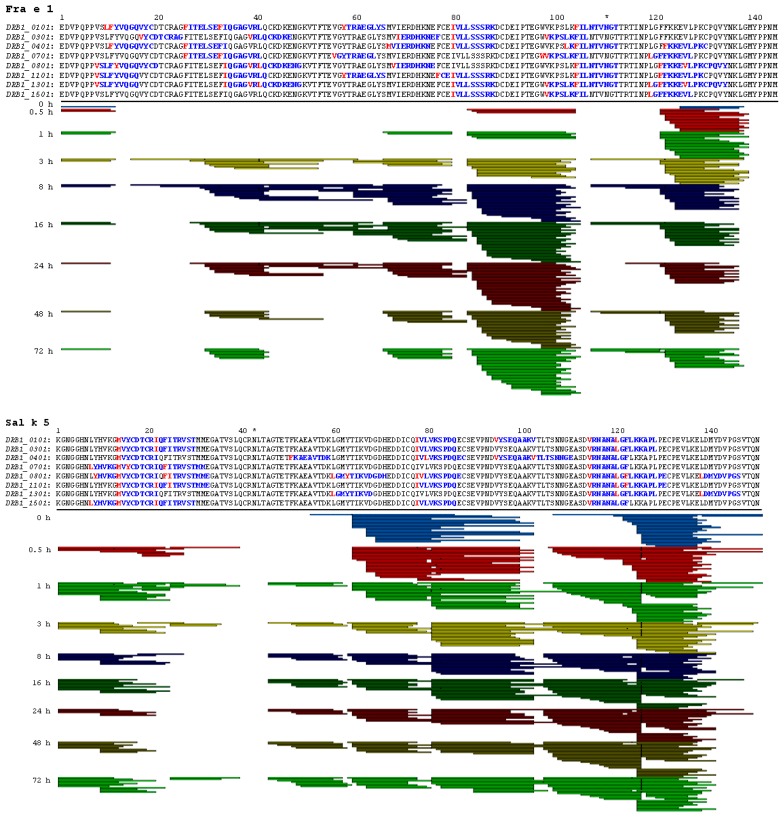

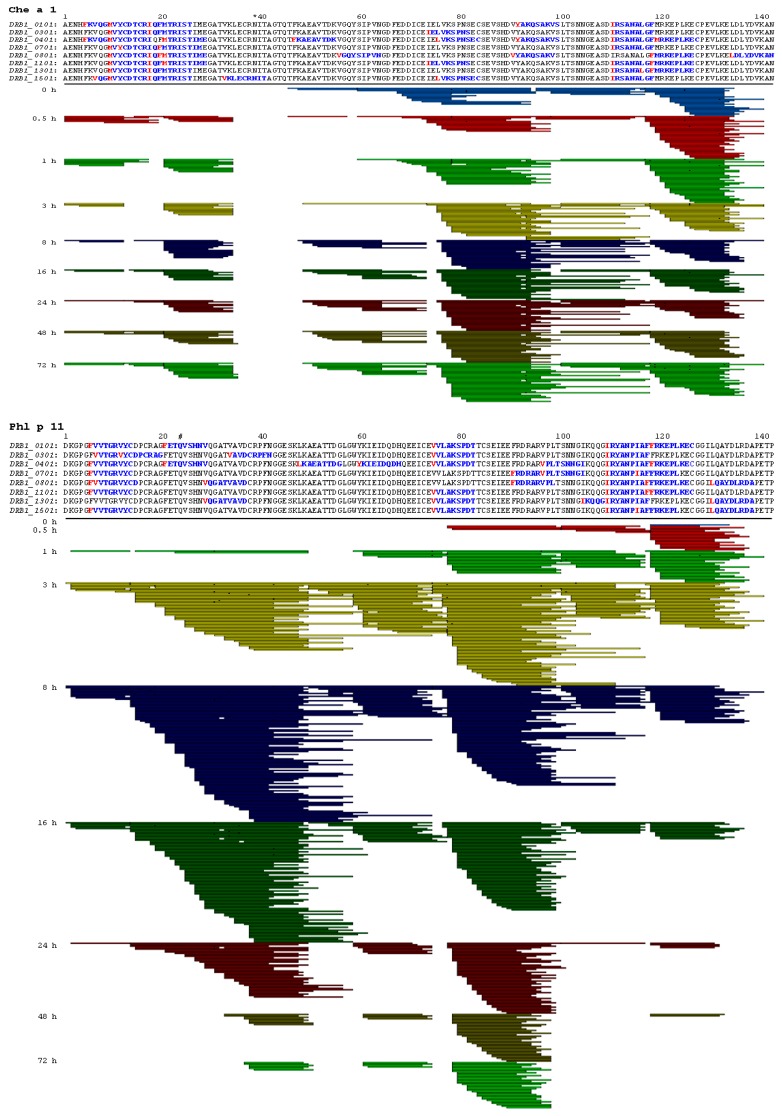

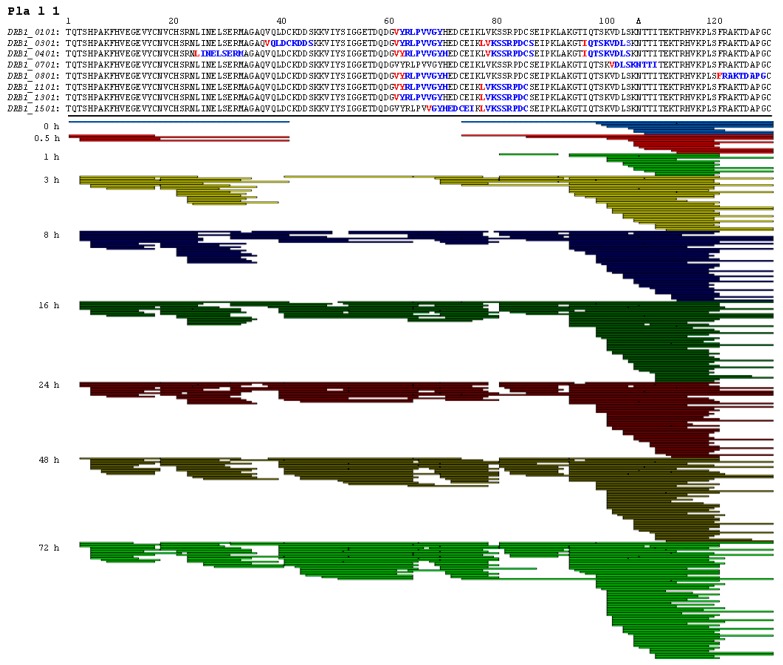
Endolysosomal degradation pattern and T cell epitopes of Ole e 1-like allergens. T cell epitopes of eight *HLA-DRB1* alleles were predicted using the ProPred software and are shown in blue; the cleavage sites are highlighted in red. Peptide patterns obtained upon endolysosomal degradation are depicted from 0–72 h of incubation. #, glycosylation site lacking glycans due to amino acid exchange; *, glycosylation site with yeast-specific glycans; Δ, glycosylation site without glycans due to expression in *E. coli*.

**Figure A2:**
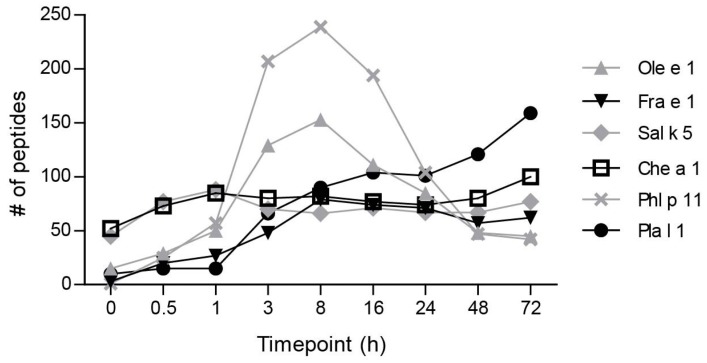
Total number of different peptides generated upon endolysosomal degradation.

Detailed peptide patterns obtained by mass spectrometry and predicted T cell epitopes for the eight most common *HLA-DRB1* alleles are shown in Figure 2 and Figure A1. Endolysosomal digestion of Phl p 11 resulted in the generation of the highest number of peptides especially between 3 and 16 h of incubation (Figure A2). Except for Ole e 1 and Phl p 11, which showed a peak around 8 h of digestion, peptide numbers were fairly comparable among the other proteins and typically increased with time of proteolysis. Analyses of the peptide pattern enabled identification of clusters potentially corresponding to regions harboring T cell epitopes (Figure 2 and Figure A1). Several dominant clusters were observed for each protein, and generally, vast overlaps with the homologous protein from the same botanical family were observed. Clusters appeared more frequently between the center and C-terminus of the proteins, while Phl p 11 showed an additional peptide cluster near the N-terminal region (Figure A1). As an example, the patterns of Ole e 1 were quite diverse, but distinct regions between residues 83–104, 105–121 or 122–139 were clearly detectable (Figure 2). Notably, the experimentally-determined major and minor T cell epitopes were perfectly localized within the dominant peptide clusters (Figure 2).

### 2.2. Structural Modeling and Molecular Dynamics Simulation

**Figure A3:**
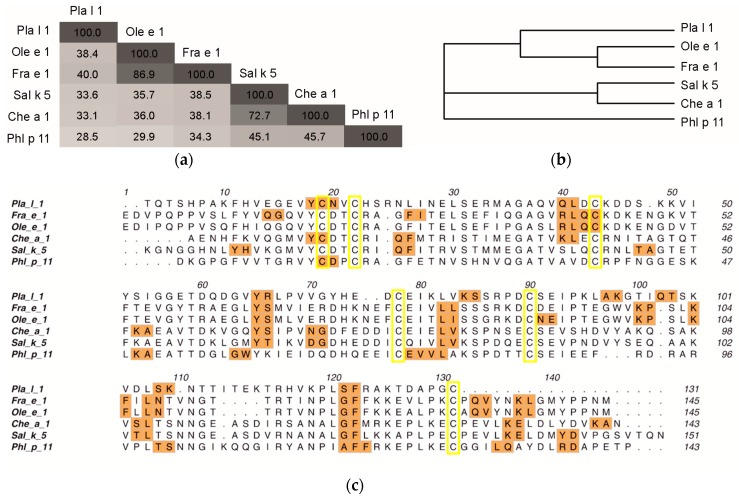
(**a**) Pairwise identity score matrix and (**b**) phylogenetic tree of Ole e 1-like sequences. Multiple sequence alignment was performed in Clustal Omega (1.2.1). Sequences were retrieved from the UniProt database considering the amino acid differences as listed in Table 1; (**c**) Alignment of all six allergens; yellow boxes indicate conserved cysteine residues, and endolysosomal cleavage sites are indicated in orange.

**Figure A4:**
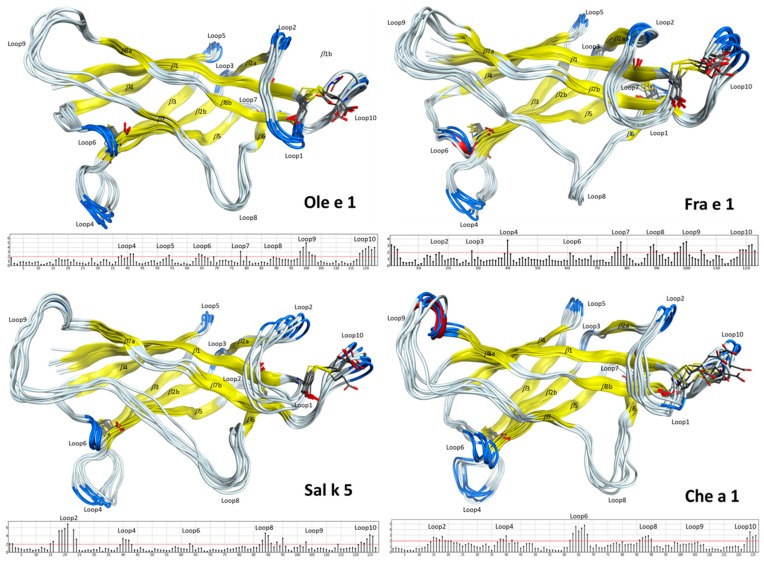

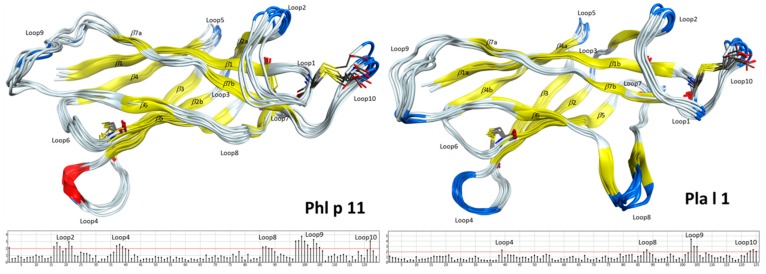
Superposition of six secondary structures (represented by ribbons) after each 100 ns of molecular dynamics simulation. β-sheets are colored yellow, flexible loops white and β-turns blue. Red designates α-helixes. The scale on the bottom shows the average RMSD (root mean square distance) value between the Cα atoms.

Two different software analyses (Swiss Model server and MOE2016.08) were used to generate homology models of the investigated Ole e 1-like proteins. Although the sequence identity between Pla l 1 and the other allergens was low (Figure A3a,b), homology models could readily be built. The resulting secondary structures were in good agreement and also displayed significant similarity with Pla l 1 (Figure A4). The modeling showed that all six allergens presented the same β-barrel fold, stabilized by three disulfide bridges, analogous to Pla l 1. Molecular dynamics simulations using NWChem pinpointed the highly flexible regions, which were localized on the random coils (Figure A4). The largest difference was observed for Phl p 11, where the hairpin loop between residues 90–99 is completely missing, while the flexible loops around residue numbers 40, 70 and 105 are more extended.

### 2.3. Endolysosomal Cleavage Sites

**Figure 3:**
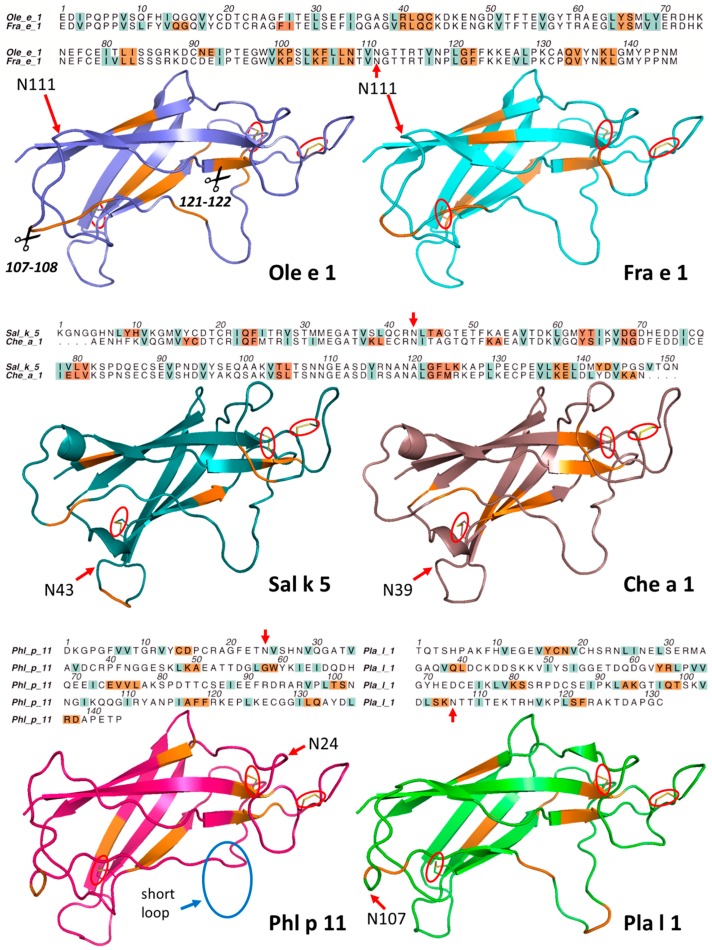
Endolysosomal cleavage sites of Ole e 1-like allergens. Dominant endolysosomal cleavage sites in the primary and secondary structure are marked in orange. Hydrophobic residues (V, I, L) in the sequence alignment are colored blue in order to point out possible P2 sites. Red arrows indicate glycosylation sites in the natural molecules, and red circles designate disulfide bridges, while the blue circle refers to the short loop region of Phl p 11.

To gain further insight, preferred proteolytic cleavage sites from endolysosomal degradation were identified and assigned to the primary and secondary structure of the proteins (Figure 3). Owing to 86.9% sequence identity, Ole e 1 and Fra e 1 showed a very similar degradation pattern. Seven identical cleavage sites were observed, co-localizing in the sequence and fitting well with the secondary structure. However, despite the high sequence identity, there were some variations in endolysosomal degradation especially at the first third of the protein. One major difference is the cleavage site at residue numbers 91–92, where we could observe a non-conservative amino acid difference in the sequence. While Ole e 1 has a polar, but overall neutral asparagine at position 91, Fra e 1 has a negatively-charged aspartic acid at that position. In the case of Sal k 5 and Che a 1, which have a sequence identity of 72.7%, there are three common cleavage sites in the flexible loops and three in the β-sheets, although two of them are rather shifted in their position. An overview of endolysosomal cleavage sites of all investigated molecules is provided in Figure A3c.

Generally, degradation patterns of Phl p 11 differed most from the other five Ole e 1-like proteins. A unique feature of Phl p 11 is the lack of the flexible 90–99 hairpin loop, while other loops are slightly longer. These local structural divergences might have caused the observed dissimilar degradation profiles. The common cleavage sites are at residue numbers 75, 100 and 118, and we observed a fast stepwise degradation behind each of them. The first scission in Phl p 11 might be between Thr100 and Ser101 at the only accessible flexible loop. Pla l 1, on the other hand, showed a similar cleavage pattern as Ole e 1, but presented an additional cleavage site between residues 95 and 96. Like the other five Ole e 1-like allergens, Pla l 1 is also cleaved around residues 37, 62, 80, 105 and 121. Nevertheless, Pla l 1 was found to possess accessible cleavage positions in two different flexible loops (cleavage between 105 and 106 and 95 and 96, respectively).

### 2.4. Substrate Binding Studies

**Figure A5:**
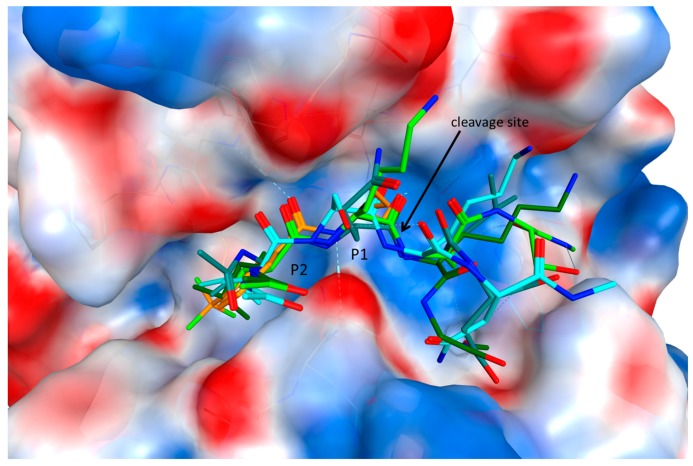
Superposition of four docking complexes with the cathepsin S inhibitor structure (3OVX). Cathepsin S and its catalytic pocket are represented as the electrostatic surface map, and the inhibitor is depicted as orange sticks. The docked tetrapeptides (green, olive and light blue sticks) correspond to four relevant P2–P2′ cleavage sites of Pla l 1. They comprise the residues 38–41 (olive sticks), 79–82 (light green sticks), 92–95 (dark green sticks) and 104–107 (light blue sticks). In all cases, the carbonyl group of the scissile peptide bond is pointing towards the oxyanion hole of cathepsin S.

Since cathepsin S was previously identified as one of the major components of the endolysosomal protease cocktail, this enzyme was further employed for in silico binding and cleavage studies [18]. The substrate specificity of cathepsin S is rather broad, but a distinct preference for small, hydrophobic, branched residues (e.g., leucine, valine or isoleucine) is noted in P2 (Table A1). In addition, small or polar unbranched residues are somewhat preferred as P1 and P1′ residues. The goal of the docking simulations was to find a substrate binding complex between cathepsin S and Ole e 1, which corresponds to the cleavage pattern observed upon endolysosomal degradation. In order to validate the docking method, first the docking complexes with the P2–P2′ tetrapeptide substrate segment cutouts from Pla l 1 were superposed on the crystallographically-determined peptide inhibitors in complex with cathepsin S (PDB Codes 3OVX [25], 1MS6 [26], 4P6G [27]) (Figure A5).

**Figure 4:**
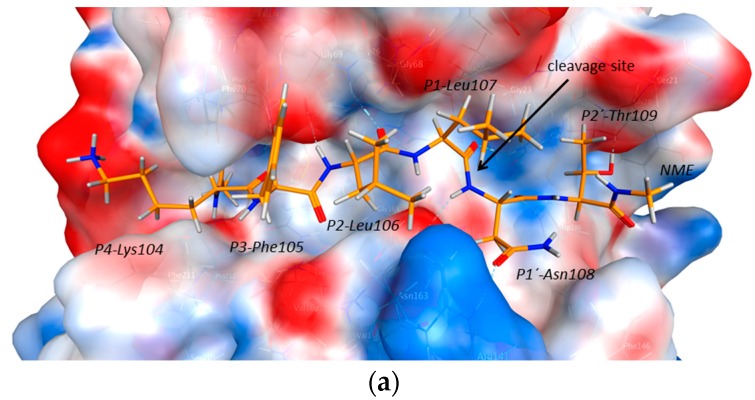

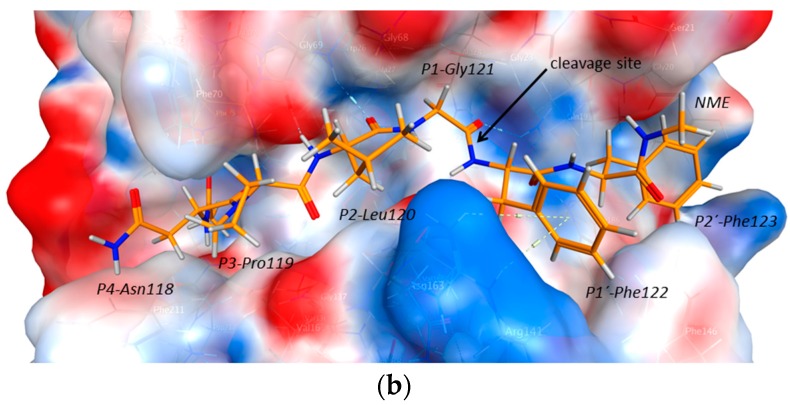
Best docking hits of hexamer peptide (P4–P2′) cut-outs of Ole e 1 at residues 104–109 (**a**) and residues 118–123 (**b**). For docking, the substrate was capped by acetyl (ACE) and N-methyl (NME) at the N and C termini to maintain the neutrality of the peptide chain. The catalytic pocket of cathepsin S is represented by the electrostatic surface, and the substrate is depicted as orange sticks.

**Figure 5:**
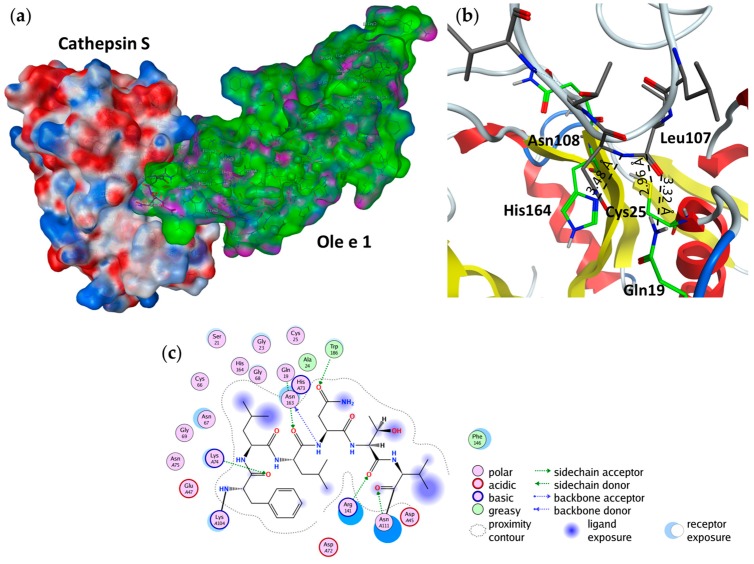
Possible cathepsin S–Ole e 1 complex. (**a**) Cathepsin S is represented by the electrostatic surface and Ole e 1 as the semi-transparent surface model with a coloring scheme indicating hydrophobic (green), mildly polar and hydrogen bonding regions (purple). Leu105 and Asn106 of the flexible loop were placed as P1 and P1′ residues into the active site, respectively; (**b**) Zoom-in of the active site region of the docking complex. Receptor carbons are green, and substrate carbons colored grey. Ole e 1 and cathepsin S are represented as ribbons; (**c**) Ligand interaction map of the P2–P1–P1′–P2′ region between cathepsin S and Ole e 1.

**Figure A6:**
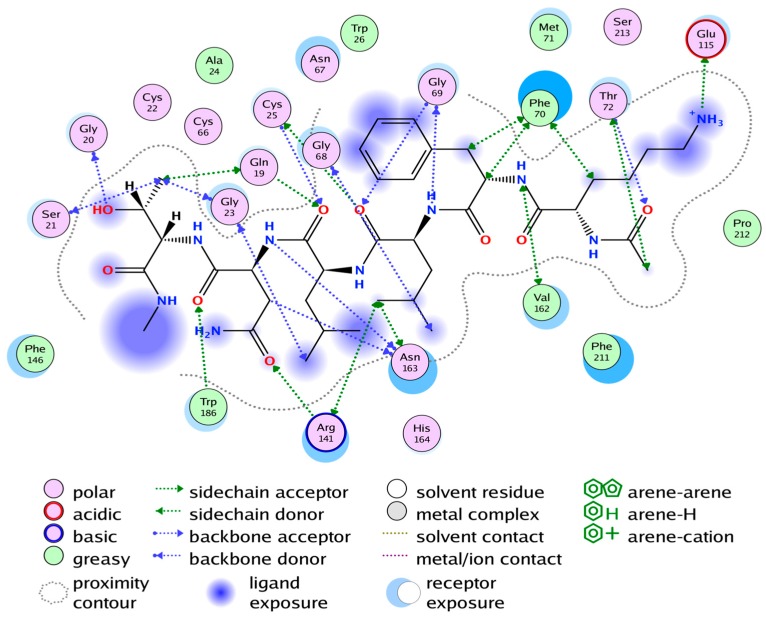
Ligand interaction map of the hexamer cutout of Ole e 1 (residues 104–109) and cathepsin S complex as generated by MOE2016.08. Dashed lines indicate hydrogen bond interactions.

As demonstrated in docking experiments with Ole e 1-derived hexapeptides (representing the P4–P2′ residues as the substrate), the carbonyl of the scissile peptide bond is pointing towards the oxyanion hole and is strongly coordinated by Gln19 and Cys25 of cathepsin S, and at the same time, the P1′ amide nitrogen is H-bonded to Asn163 (Figure 4 and Figure A6). The P2 leucine residue has a strong backbone interaction with Gly69, and its side chain is buried in the hydrophobic pocket of the enzyme. The P3 phenylalanine is building a π-stacking with Phe70, and the amide nitrogen is coordinated by Val162. The side chain of P4 lysine is hydrogen bonded to Glu115 and the substrate backbone (of ACE) to Thr72. On the prime side of the cleavage site, the P1′ Asn residue is coordinated by Arg141 and Trp186, and the P2′ threonine is interacting with Gly20. This way, both the short and the long-range interactions could be reproduced. In addition, in order to simulate the first cleavage step at loop 105, which is one of the most accessible flexible loops that can be processed by the enzyme, we generated a possible complex of cathepsin S and Ole e 1 (Figure 5), where the P1 and P1′ residues of Ole e 1 were Leu107 and Asn108, respectively. In this complex, the carbonyl oxygen of Leu107 is buried in the oxyanion hole, and the scissile peptide bond is ideally positioned for a nucleophilic attack of Cys25. ND1(His164) is also within H-bond distance (3.48 Å) to the backbone amide nitrogen of Asn108. Once loop 105 is cleaved, the protein might be able to unfold, and the β-sheets become further accessible for subsequent digestion.

### 2.5. Cathepsin S Inhibition

**Figure 6:**
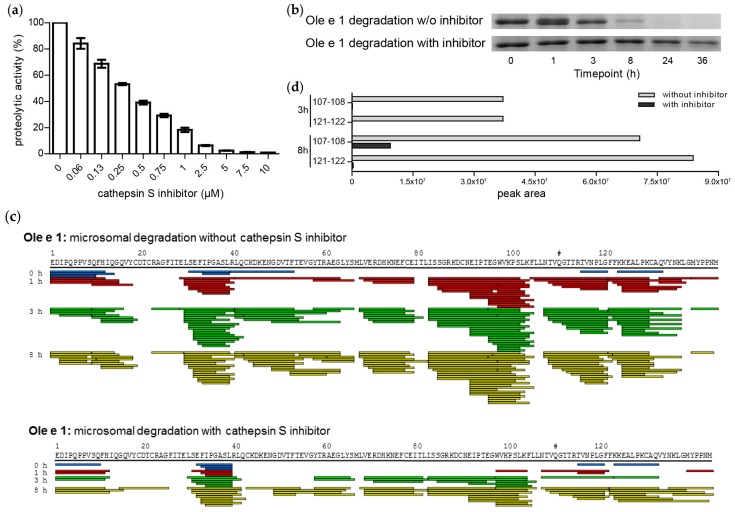
Cathepsin S inhibition experiments using Z-FL-COCHO. (**a**) Fluorogenic substrate (Z-VVR-AMC) activity of endolysosomal was decreased by cathepsin S inhibitor; (**b**) Endolysosomal proteolysis of Ole e 1 using Z-FL-COCHO as the inhibitor; (**c**) Comparison of the Ole e 1 degradation pattern with and without cathepsin S inhibition; (**d**) Mass spectrometry-based peak areas of peptides flanking the Ole e 1 cleavage sites 107–108 and 121–122 obtained upon proteolysis with and without cathepsin S inhibitor at 3 and 8 h, respectively.

In order to verify the relevance of cathepsin S proteolysis during endolysosomal degradation, we inhibited its reactivity using the specific inhibitor Z-FL-COCHO. First, the optimal inhibitor concentration was determined using a fluorogenic substrate revealing that 10 µM inhibitor reduced cathepsin S reactivity to ~1% (Figure 6a). Subsequently, Ole e 1 was digested in the presence of 10 µM inhibitor, which dramatically reduced the proteolytic activity of the endolysosomal cocktail (Figure 6b). In contrast to the control digest (without inhibitor) leading to substantial degradation within 8 h, considerably less reactivity starting with degradation at 36 h was noted in the cathepsin S inhibited digest. Cathepsin S inhibition also led to more restricted peptide diversity while the overall pattern remained similar, potentially due to residual protease activity (Figure 6c). However, semi-quantitative analysis of peptide peak areas obtained by mass spectrometry showed an 8–28-fold reduction in detectable total peptide amount during digestion. We further investigated two potentially relevant cathepsin S cleavage sites at 107–108 and 121–122 in more detail. The sum of peptides flanking either of the two proteolytic sites demonstrated that cathepsin S inhibition significantly reduced the peptide amounts generated after 3 and 8 h of digestion (Figure 6d).

## 3. Discussion

Knowledge of proteolytic stability is important information relating to the immunogenicity of proteins. Despite the fact that Ole e 1-like allergens are considered relevant allergens, research has mostly focused on their IgE reactivity. Data on immunogenicity and T cell epitopes are limited and only available for Ole e 1 [17]. Endolysosomal degradation is a straightforward in vitro method to obtain information on protein immunogenicity while it also enables identification of regions harboring T cell epitopes [18,28,29,30]. Within this study, we evaluated the endolysosomal stability and degradation of six allergenic members of the Ole e 1-like protein family originating from pollen of tree, grass and weed species, all produced as recombinant proteins. Production of Ole e 1-like proteins is typically achieved by secreted expression in *Pichia pastoris* [5,31,32,33]. While good expression yields of IgE reactive molecules were obtained in yeast, Ole e 1-like proteins can present heterogeneous N-termini and/or host-specific glycans unless glycosylation sites were removed [5,8,31,32,33]. Taking this into account, folded Pla l 1 was recently produced in *E. coli* using the strain Rosetta-gamiB pLysS to enable disulfide bond formation [2]. Concerning mass spectrometry-based peptide analysis, the use of recombinant non-glycosylated molecules is favored. Thus, isoforms with different sequences in the case of natural molecules and heterogeneous glycan moieties interfering with qualitative and quantitative results can be avoided.

Even though sequence identities are considerably low between some members of the Ole e 1-like protein family, similar peptide profiles were observed upon endolysosomal degradation. However, degradation kinetics varied considerably with high susceptibility for Phl p 11, while Pla l 1 was extremely stable until 72 h of incubation. Ole e 1 and Fra e 1 are considered the most relevant primary allergy sensitizers within this protein family [5,10]. Interestingly, they showed intermediate degradation kinetics, with substantial proteolysis between 3 and 8 h. This observation supports the hypothesis that proteins with medium stability seem to be the most efficient for antigen presentation and immunogenicity in the course of allergic sensitization [22].

A number of mapping algorithms and web tools for the prediction of T cell epitopes have become available during the past few years. T cell peptides are bound to MHC class II molecules in a linear manner, and while the binding grooves are open at both ends, a peptide length of nine amino acids has been found to be critical for binding [34]. For our study, the online tool ProPred was used to predict T cell epitopes with a length of nine amino acids. Indeed, we observed that predicted peptides were typically localized within peptide clusters identified by endolysosomal degradation. Prediction of T cell epitopes is highly dependent on the software and *HLA* allele selection; thus, the interpretation of results can be challenging [35]. While the majority of predicted epitopes were co-localizing with identified peptide clusters, we observed some differences for Pla l 1, showing a dominant cluster in the C-terminal region, while predicted epitopes in this region were scarce.

Notably, Ole e 1 peptide clusters revealed in this study perfectly corresponded to the three previously identified immunodominant T cell epitopes 91–102, 109–120 and 119–130 [17]. Additionally, we observed matches for T cell peptides with slightly lower responses, i.e., 64–75 and 129–140. While T cell epitope identification using synthetic peptides is presently considered the most reliable technique, typically numerous overlapping peptides are required to analyze the entire sequence of a molecule. In addition, there is a need to obtain patient samples for analysis, which requires a certain number of subjects to ensure diversity. Some studies have demonstrated the vast overlap of T cell epitopes and peptide clusters identified by endolysosomal degradation [22,28,29,30]. Thus, we consider the endolysosomal degradation assay a valuable tool for, first, identification and potentially narrowing down the number of synthetic peptides required for an experimental setup.

To localize cleavage sites within the secondary structure of the proteins, homology models based on Pla l 1 were generated, all showing the conserved β-barrel core structure. Proteins were assembled into four botanical families, and similar degradation patterns were observed for the homologous allergens. Ole e 1 and Fra e 1 demonstrated highly similar patterns even though the latter molecule is *N*-glycosylated at one site, which prevented the identification of the corresponding glycopeptide in mass spectrometry. However, there were some minor differences in cleavage patterns of the N-terminal region, albeit amino acid residues were conserved. Similar patterns were also observed for the homologous molecules Sal k 5 and Che a 1. Potentially, there is an additional cluster around 35–45, which could not be revealed due to glycosylation at residue 43 and 39, respectively. Even though the sequence similarity of Pla l 1 and other family members is only 28.5–40.0%, rather similar degradation patterns were observed. In contrast, Phl p 11 demonstrated significant differences in comparison with the other five Ole e 1-like proteins, which might be due to the large discrepancies in the flexible regions. In particular, the hairpin loop around 90–99 is missing, while other loop regions are longer compared to the homologs. Interestingly, all tested allergens agreed on the cleavage sites around residue numbers 100 and 120, positions located in one of the most accessible flexible loops.

Various endo- and exo-proteases, e.g., cathepsins and legumain, are responsible for peptide generation in the endolysosome and are thus enrolled in the in vitro degradome assay. It was shown that cathepsin S represents one of the major proteolytic components in endolysosomal cell fractions [23,24]. In a previous study, 8/13 peptide clusters were congruent when using purified cathepsin S compared to endolysosomal protease preparations from human or murine DCs and the JAWS II cell line, respectively [18]. To unequivocally determine the contribution of cathepsin S cleavage, we specifically inhibited its proteolytic activity in the endolysosomal cocktail. Indeed, we demonstrated that cathepsin S inhibited digests showed considerably lower activity using a fluorogenic substrate, but more importantly also when using Ole e 1 for digestion.

Due to its relevance during endolysosomal processing, cathepsin S was chosen for in silico binding and cleavage studies. Even though the substrate specificity of cathepsin S, as well as other lysosomal cysteine endopeptidases is quite broad, there is clear preference for leucine, valine and isoleucine residues at P2 according to the MEROPS Peptidase Database [36]. Sequence analyses of the degradation assay confirmed that the detected cleavage sites indeed mostly presented a leucine or a valine as the P2 residue. The crystal structure of human cathepsin S was previously determined by McGrath et al., and thereby, the catalytic and substrate binding residues could be identified [37]. The authors point out that the S1 substrate binding pocket is determined mainly by the oxyanion hole, which is formed by Gln19 and Gly23. The S1′ catalytic pocket comprises the residues Asn158, Trp177, His159 and Ala136, which corresponds to Asn163, Trp26, His164 and Ala124 in our structure. The S2 pocket is formed by hydrophobic residues, which also explains the only substrate specificity of the enzyme. In order to validate the docking method, we first performed in silico binding studies of the proteolytic sites between Pla l 1 cutouts of P2–P1–P1′–P2′ and the enzyme cathepsin S. Afterwards, the resulting complexes were superimposed on the crystal structures of the enzyme inhibitor complex of cathepsin S (PDB Codes 3OVX [25], 1MS6 [26], 4P6G [27]). They were found to be in good agreement regarding the position of the P1 carbonyl and P1, P2 subsites.

Ole e 1 was used as a representative model allergen to further analyze two distinct cleavage sites conserved among all tested models. Based on the obtained peptide clusters and the conserved location in the loop, an early cathepsin S cleavage site might be located at positions 107–108 (numbering according to Ole e 1). The complex of cathepsin S and intact Ole e 1 at this position represents the most likely possibility, as other cleavage sites were less accessible to the protease. The finding is supported by the fact that corresponding peptides were detected starting from 0.5 h of digestion and non-cleaved peptides spanning this area could not be observed in mass spectrometry. Further analysis using a cathepsin S inhibitor verified the specificity of the cleavage site, as substantially lower peptide amounts were detected upon inhibition.

Once the extended loop is cleaved, Ole e 1 might be more accessible for further cleavage sites located in, e.g., the β-barrel of the protein. Notably, this region also corresponds to the immunodominant T cell epitopes identified for Ole e 1 [17]. Further supporting the idea that the first cleavage event is localized in this loop is the fact that cathepsin S is stable in a broad pH range. Thus, it represents an enzyme that is already active in less acidic pH, and thus, involved in early recognition of substrates [38,39]. Despite the sequence divergence among members of the Ole e 1-like protein family, the T cell epitopes might be located in similar regions as a consequence of structural accessibility to endolysosomal proteases. Interestingly, the glycan moiety of Ole e 1, Fra e 1 and Pla l 1 is also found within this protein region, while residual molecules are glycosylated in different loops. Another potential cleavage site in the loop region at 104–105 was also investigated. However, results of docking experiments were less convincing, and peak areas of these peptides were substantially lower compared to those flanking the cleavage sites 107–108 and 121–122.

## 4. Materials and Methods

### 4.1. Recombinant Production of Ole e 1-Like Allergens

Recombinant Ole e 1, Fra e 1, Sal k 5, Che a 1 and Phl p 11 were purified from *Pichia pastoris* according to published protocols [2,5,14,31,32]. Recombinant Pla l 1 was obtained from *Escherichia coli* expression as recently published [2]. Details on physico-chemical and immunological characterization are found in Stemeseder et al. and a summary of Ole e l-like allergens investigated in this study is provided in Table 1. Sequence alignment was performed with Clustal Omega (1.2.1) (UCD, Dublin) [40].

### 4.2. Amino Acid Analysis

Amino acid distribution and protein concentration of the purified proteins were determined using the Pico-Tag method (Waters, Milford, MA, USA). Briefly, 10 µg of the protein were hydrolyzed with 10 M HCl and derivatized with phenyl isothiocyanate. Phenylthiocarbamyl amino acid derivatives were analyzed by reversed phase high-performance liquid chromatography (UltiMate 3000, Thermo Fischer, Waltham, MA, USA), using a 3.0 × 150 mm XSELECT™ HSS T3 3.5-µm column (Waters, Dublin, Ireland). Hydrolyzed amino acid peaks were quantified at 254 nm by peak area comparison to amino acid standard H (Pierce, Rockford, IL, USA).

### 4.3. Endolysosomal Degradation Assay

The proteolytic stability to endolysosomal proteases was investigated using the endolysosomal degradation assay. Endolysosomes were isolated from murine JAWS II dendritic cells by differential centrifugation as previously described [18]. Briefly, 5 × 10^7^ cells were resuspended in 10 mM Tris/acetate pH 7.0 containing 250 mM sucrose and homogenized on ice using a glass Dounce tissue grinder (Sigma-Aldrich, St. Louis, MO, USA). The cell homogenate was centrifuged for 10 min at 6000× *g* to obtain a post-nuclear supernatant. After an ultracentrifugation step for 60 min at 100,000× *g*, the microsomal content was released by five freeze/thaw cycles on liquid nitrogen and room temperature, respectively, and subsequently stored at −20 °C. Allergens (5 µg) were incubated with 7.5 µg of isolated microsomal proteins in a final volume of 20 µL containing 100 mM citrate buffer pH 4.8 and 2 mM dithiothreitol; the proteolytic digestion at 37 °C was monitored up to 72 h. Reactions were stopped by denaturation at 95 °C for 5 min at the time points of 0, 0.5, 1, 3, 8, 16, 24, 48 and 72 h, and samples were stored at −20 °C until further use.

### 4.4. Cathepsin S Inhibition Assays

An enzymatic assay was carried out to assess the concentration of cathepsin S inhibitor needed to inhibit the cathepsin S activity in the microsomal fraction. Microsomal fractions (375 µg/mL) were pre-incubated with series (0–10 µM) of cathepsin S inhibitor, Z-FL-COCHO (Sigma-Aldrich), in assay buffer (100 mM citrate pH 4.8, 2 mM DTT) at 25 °C for 15 min. The enzymatic reaction was performed using 50 µM cathepsin S fluorogenic substrate, Z-VVR-AMC (Bachem, Bubendorf, Switzerland). Reactions were carried out in a 50-µL reaction volume in a 96-well black flat bottom assay plate (Corning Inc., Corning, NY, USA) at 37 °C. The fluorescence signal was recorded using a Tecan Infinite 200 plate reader (Tecan, Männedorf, Switzerland) at an excitation wavelength of 380 nm and emission wavelength of 460 nm for 15 min of continuous measurement with a 30-s interval reading. A cysteine protease inhibitor E64 (Sigma-Aldrich) was used as a control.

To determine the relevance of cathepsin S in endolysosomal proteolysis, degradation of Ole e 1 was monitored from 0–36 h using the inhibitor Z-FL-COCHO at a concentration of 10 µM. A control assay without inhibitor was performed in parallel, and experimental conditions were performed as described above in the section Endolysosomal Degradation Assay.

### 4.5. Gel Electrophoresis

Degradation of intact proteins was monitored by reducing sodium dodecyl sulfate polyacrylamide gel electrophoresis (SDS-PAGE) using 15% gels. Proteins were visualized by staining with Coomassie Brilliant Blue R-250 (Bio-Rad, Hercules, CA, USA). The percentage of degradation was measured as intensity of protein bands using Image Lab 4.0.1 (Bio-Rad).

### 4.6. T Cell Epitope Prediction

Using the web tool ProPred, T cell epitope predictions were performed [41]. ProPred predicts MHC class II binding regions in an antigen sequence using quantitative matrices. Our analyses focused on eight common alleles (DRB1*0101, DRB1*0301, DRB1*0401, DRB1*0701, DRB1*0801, DRB1*1101, DRB1*1301 and DRB1*1501), which are considered representative for the human population [42]. Top scoring peptides were obtained by selection of peptides with a threshold of 5%.

### 4.7. Mass Spectrometry

For mass spectrometry of peptide fragments generated by endolysosomal proteolysis, 0.5 µg of digested Ole e 1-like proteins were desalted using C_18_ ZipTips (EMD Millipore, Billerica, MA, USA) and separated by reverse-phase nano-HPLC (Dionex Ultimate 3000, Thermo Fisher Scientific, Bremen, Germany, column: PepSwift Monolithic Nano Column, 100 μm × 25 cm, Dionex, Sunnyvale, Ca, USA). The column was eluted with an acetonitrile gradient (Solvent A: 0.1% (*v*/*v*) formic acid, 0.01% (*v*/*v*) trifluoroacetic acid, 5% (*v*/*v*) dimethyl sulfoxide; solvent B: 0.1% (*v*/*v*) formic acid, 0.01% (*v*/*v*) trifluoroacetic, 90% (*v*/*v*) acetonitrile, 5% (*v*/*v*) dimethyl sulfoxide; 5–45% B in 60 min) at a flow rate of 1 μL/min at 55 °C. Peptides were analyzed with a Q Exactive Orbitrap mass spectrometer (Thermo Fisher Scientific) directly coupled to the HPLC. Capillary voltage at the nano-electrospray head was 2 kV, and the instrument was tuned for maximum sensitivity. For peptide assignments, a top 12 method was used with a normalized fragmentation energy at 27%. Survey and fragment spectra were analyzed with Proteome Discoverer Version 1.4 (Thermo Fisher Scientific) with SequestHT as the search engine and PEAKS Studio 8 (Bioinformatics Solutions, Waterloo, ON, Canada). Searches were done with single allergen sequences, considering post-translational modifications and amino acid exchanges as determined by MS/MS (Table 1). Only peptides with high confidence scores (XCorr ≥ 2.3 for SequestHT, −10lgP ≥ 35 for PEAKS) were considered. Protein identity was confirmed prior to the degradome analyses by tryptic digestion (ProteoExtract All-in-One Trypsin Digestion Kit (EMD Millipore) and MS/MS analysis as described above. Parallel to sequence analysis, the PTM and Spider modules of PEAKS Studio were used to identify post-translational modifications and amino acid exchanges, respectively. Protein degradation patterns were generated and visualized using the online tool Draw Map [43]. For semi-quantitative analysis of Ole e 1 peptides obtained with and without cathepsin S inhibitor, mass spectrometry-based peak areas of peptides flanking the investigated cleavage sites at 107–108 and 121–122 were summed up.

### 4.8. Homology Modeling and Molecular Dynamics

The crystal structure of Pla l 1 (PDB Code 4Z8W) was used as a template to generate the secondary structures [2]. Homology models of Ole e 1, Fra e 1, Sal k 5, Che a 1 and Phl p 11 were created by two different approaches. On the one hand, we used the Swiss Model server [44], and on the other hand, the software packet MOE2016.08 [45]. N and C terminal extensions of all allergens in comparison with Pla l 1 were automatically truncated by both modeling programs. The newly-generated homology models were aligned with Pla l 1 using the online server Clustal Omega [40], and the alignment was graphically formatted with Aline [46]. For structural superposition, MOE2016.08 was used [45]. To perform the molecular dynamics calculations in a solution environment, similar to that in the in vivo environment, each allergen was first titrated at pH 4.8 (experimental pH) using the Protonate 3D function of MOE2016.08 [45]. The resulting protein was solvated in an 80 Å cubic box of water, and counter ions (either Na^+^ or Cl^−^) were added to maintain overall neutrality of the protein. Afterwards, a series of equilibration steps was carried out by performing molecular dynamics annealing runs for 100 ps at temperatures of 50 K, 150 K, 200 K and 250 K and for 300 ps at 298.15 K. The molecular dynamics calculations were accomplished using the AMBER99 force field as implemented in NWChem 6.6 (PNNL, Richland, WA, USA) [47].

### 4.9. Structure Preparation and Docking

Protonation of the residues was carried out using the Protonate 3D function at pH 4.8 as implemented in MOE2016.08 [45] for both cathepsin S and allergens, respectively. The structure of cathepsin S was retrieved from the Protein Database [48], PDB Code 4P6G [27]. The choice of the tetrapeptide and hexapeptide amino acid chains for the docking procedure was based on the results of degradation experiments. In the case of the tetrapeptides, the P2–P1–P1′–P2′, peptides were cut out of Pla l 1, and in the case of Ole e 1, hexapeptides as P4–P2′ residues were applied. In all cases, the peptides were terminated by ACE (acetyl) at the N-terminus and NME (N-methyl) at the C-terminus to maintain the peptide-like structure and neutrality of the substrate.

The docking simulations were performed using the following settings of the software package of MOE 2016.08 [45]. In the potential energy setup panel, AMBER99 was chosen as the force field. Three placement methods, α triangle, triangle matcher (both general docking) and protein-protein docking, were employed to find the optimal docking hits. In the case of the α triangle and triangle matcher placement, the scoring was the London dG method, and force field refinement was applied allowing for the flexibility of the catalytic site within 5.0 Å. Each run was adjusted to retain 30 docked conformations as a cut-off unless less suitable poses were found. The top poses were retained for further analysis, investigating the H-bond distances between the substrate backbone and cathepsin S. For cathepsin S, the residues Gln19, Cys25, Gly69, Asn163 and His164 were defined as the binding pocket. The rotate bond of the Docking Simulation panel was enabled for the general docking. The best docking hits were optimized using the energy minimization function of MOE2016.08 [45] with the AMBER99 force field method.

## 5. Conclusions

The use of a dendritic cell line for in vitro endolysosomal degradation enabled identification of proteolytic cleavage sites in allergenic Ole e 1-like proteins. Despite their sequence divergence, the proteins were rather congruent regarding their peptide profiles and proteolytic accessibility within the large flexible loop. The endolysosomal degradation assay provided, first, information on immunogenicity, as well as potential T cell epitopes in members of the Ole e 1-like protein family.

## Figures and Tables

**Table 1:** Recombinant Ole e 1-like allergens investigated for endolysosomal degradation.

Allergen	Plant	Accession Number	Host Organism	Protein Modification
Ole e 1.0101	*Olea europaea*	P19963.2	*P. pastoris*	A99V, K106L, N111Q, deamidation, oxidation
	Olive			
Fra e 1.0101	*Fraxinus excelsior*	AAQ08947.1	*P. pastoris*	minor deamidations, yeast-specific glycosylation at N111
	Ash			
Sal k 5.0101	*Salsola kali*	ADK22842.1	*P. pastoris*	K3N, G84D, I91V, deamidation, yeast-specific glycosylation at N43
	Russian thistle			
Che a 1.0101	*Chenopodium album*	Q8LGR0.1	*P. pastoris*	deamidation, methionine oxidation, yeast-specific glycosylation at N39
	Lamb’s quarters			
Phl p 11.0101	*Phleum pratense*	Q8H6L7.1	*P. pastoris*	N24Q
	Timothy grass			
Pla l 1.0101	*Plantago lanceolata*	P82242.2	*E. coli*	None
	English plantain			

## Abbreviations

ACE	Acetyl
DC	Dendritic cell
HCl	Hydrochloric acid
MHC	Major histocompatibility complex
NME	N-methyl
SDS-PAGE	Sodium dodecyl sulfate polyacrylamide gel electrophoresis

## Appendix A

**Table A1:** Substrate specificity matrix of cathepsin S extracted from the MEROPS Peptidase Database.

Amino Acid	P4	P3	P2	P1	P1′	P2′	P3′	P4′
Ala	63	70	22	87	107	98	82	53
Arg	6	7	1	13	6	12	8	8
Asn	30	34	4	41	28	30	39	30
Asp	37	19	0	28	33	27	71	77
Cys	9	20	14	14	15	19	6	14
Gln	38	33	14	82	67	51	37	47
Glu	59	53	2	81	68	38	42	80
Gly	48	63	5	128	118	86	80	91
His	20	17	3	17	26	29	55	40
Ile	25	58	76	9	19	34	21	27
Leu	40	71	244	44	24	31	23	22
Lys	7	9	1	8	6	26	15	23
Met	20	20	55	15	10	8	2	6
Phe	30	42	69	10	14	16	22	12
Pro	42	51	5	1	0	63	106	79
Ser	51	57	11	85	128	72	51	51
Thr	22	41	17	76	40	59	39	46
Trp	10	7	11	6	4	4	8	4
Tyr	14	14	28	5	13	6	16	10
Val	48	47	172	6	24	41	27	30

Residues >75 are depicted in green.

## References

[1] Radauer C., Bublin M., Wagner S., Mari A., Breiteneder H. (2008). Allergens are distributed into few protein families and possess a restricted number of biochemical functions. J. Allergy Clin. Immunol..

[2] Stemeseder T., Freier R., Wildner S., Fuchs J.E., Briza P., Lang R., Batanero E., Lidholm J., Liedl K.R., Campo P. (2017). Crystal structure of Pla l 1 reveals both structural similarity and allergenic divergence within the Ole e 1-like protein family. J. Allergy Clin. Immunol..

[3] Batanero E., Villalba M., Rodriguez R. (1994). Glycosylation site of the major allergen from olive tree pollen. Allergenic implications of the carbohydrate moiety. Mol. Immunol..

[4] Van Ree R., Cabanes-Macheteau M., Akkerdaas J., Milazzo J.P., Loutelier-Bourhis C., Rayon C., Villalba M., Koppelman S., Aalberse R., Rodriguez R. (2000). β(1,2)-Xylose and α(1,3)-fucose residues have a strong contribution in IgE binding to plant glycoallergens. J. Biol. Chem..

[5] Barderas R., Purohit A., Papanikolaou I., Rodriguez R., Pauli G., Villalba M. (2005). Cloning, expression, and clinical significance of the major allergen from ash pollen, Fra e 1. J. Allergy Clin. Immunol..

[6] Calabozo B., Barber D., Polo F. (2002). Studies on the carbohydrate moiety of Pla l 1 allergen. Identification of a major N-glycan and significance for the immunoglobulin E-binding activity. Clin. Exp. Allergy.

[7] Villalba M., Rodriguez R., Batanero E. (2014). The spectrum of olive pollen allergens. From structures to diagnosis and treatment. Methods.

[8] Marknell DeWitt A., Niederberger V., Lehtonen P., Spitzauer S., Sperr W.R., Valent P., Valenta R., Lidholm J. (2002). Molecular and immunological characterization of a novel timothy grass (phleum pratense) pollen allergen, Phl p 11. Clin. Exp. Allergy.

[9] Castro A.J., Alche J.D., Calabozo B., Rodriguez-Garcia M.I., Polo F. (2007). Pla 1 1 and Ole e 1 pollen allergens share common epitopes and similar ultrastructural localization. J. Investig. Allergol. Clin. Immunol..

[10] Villalba M., Batanero E., Lopez-Otin C., Sanchez L.M., Monsalve R.I., Gonzalez de la Pena M.A., Lahoz C., Rodriguez R. (1993). The amino acid sequence of Ole e I, the major allergen from olive tree (*Olea europaea*) pollen. Eur. J. Biochem..

[11] Batanero E., Crespo J.F., Monsalve R.I., Martin-Esteban M., Villalba M., Rodriguez R. (1999). IgE-binding and histamine-release capabilities of the main carbohydrate component isolated from the major allergen of olive tree pollen, Ole e 1. J. Allergy Clin. Immunol..

[12] Palomares O., Swoboda I., Villalba M., Balic N., Spitzauer S., Rodriguez R., Valenta R. (2006). The major allergen of olive pollen Ole e 1 is a diagnostic marker for sensitization to Oleaceae. Int. Arch. Allergy Immunol..

[13] Villalba M., Barderas R., Mas S., Colas C., Batanero E., Rodriguez R. (2014). Amaranthaceae pollens: Review of an emerging allergy in the mediterranean area. J. Investig. Allergol. Clin. Immunol..

[14] Castro L., Mas S., Barderas R., Colas C., Garcia-Selles J., Barber D., Rodriguez R., Villalba M. (2014). Sal k 5, a member of the widespread Ole e 1-like protein family, is a new allergen of russian thistle (*Salsola kali*) pollen. Int. Arch. Allergy Immunol..

[15] Barderas R., Villalba M., Lombardero M., Rodriguez R. (2002). Identification and characterization of Che a 1 allergen from chenopodium album pollen. Int. Arch. Allergy Immunol..

[16] Gadermaier G., Eichhorn S., Vejvar E., Weilnbock L., Lang R., Briza P., Huber C.G., Ferreira F., Hawranek T. (2014). Plantago lanceolata: An important trigger of summer pollinosis with limited IgE cross-reactivity. J. Allergy Clin. Immunol..

[17] Cardaba B., del Pozo V., Jurado A., Gallardo S., Cortegano I., Arrieta I., del Amo A., Tramon P., Florido F., Sastre J. (1998). Olive pollen allergy: Searching for immunodominant T-cell epitopes on the Ole e 1 molecule. Clin. Exp. Allergy.

[18] Egger M., Jurets A., Wallner M., Briza P., Ruzek S., Hainzl S., Pichler U., Kitzmuller C., Bohle B., Huber C.G. (2011). Assessing protein immunogenicity with a dendritic cell line-derived endolysosomal degradome. PLoS ONE.

[19] Kitzmuller C., Zulehner N., Roulias A., Briza P., Ferreira F., Fae I., Fischer G.F., Bohle B. (2015). Correlation of sensitizing capacity and T-cell recognition within the Bet v 1 family. J. Allergy Clin. Immunol..

[20] Delamarre L., Couture R., Mellman I., Trombetta E.S. (2006). Enhancing immunogenicity by limiting susceptibility to lysosomal proteolysis. J. Exp. Med..

[21] Delamarre L., Pack M., Chang H., Mellman I., Trombetta E.S. (2005). Differential lysosomal proteolysis in antigen-presenting cells determines antigen fate. Science.

[22] Machado Y., Freier R., Scheiblhofer S., Thalhamer T., Mayr M., Briza P., Grutsch S., Ahammer L., Fuchs J.E., Wallnoefer H.G. (2016). Fold stability during endolysosomal acidification is a key factor for allergenicity and immunogenicity of the major birch pollen allergen. J. Allergy Clin. Immunol..

[23] Pluger E.B., Boes M., Alfonso C., Schroter C.J., Kalbacher H., Ploegh H.L., Driessen C. (2002). Specific role for cathepsin S in the generation of antigenic peptides in vivo. Eur. J. Immunol..

[24] Rudensky A., Beers C. (2006). Lysosomal cysteine proteases and antigen presentation. Ernst Schering Res. Found. Workshop.

[25] Cai J., Robinson J., Belshaw S., Everett K., Fradera X., van Zeeland M., van Berkom L., van Rijnsbergen P., Popplestone L., Baugh M. (2010). Trifluoromethylphenyl as P2 for ketoamide-based cathepsin S inhibitors. Bioorg. Med. Chem. Lett..

[26] Ward Y.D., Thomson D.S., Frye L.L., Cywin C.L., Morwick T., Emmanuel M.J., Zindell R., McNeil D., Bekkali Y., Hrapchak M. (2002). Design and synthesis of dipeptide nitriles as reversible and potent cathepsin S inhibitors. J. Med. Chem..

[27] Jadhav P.K., Schiffler M.A., Gavardinas K., Kim E.J., Matthews D.P., Staszak M.A., Coffey D.S., Shaw B.W., Cassidy K.C., Brier R.A. (2014). Discovery of cathepsin S inhibitor ly3000328 for the treatment of abdominal aortic aneurysm. ACS Med. Chem. Lett..

[28] Toda M., Reese G., Gadermaier G., Schulten V., Lauer I., Egger M., Briza P., Randow S., Wolfheimer S., Kigongo V. (2011). Protein unfolding strongly modulates the allergenicity and immunogenicity of Pru p 3, the major peach allergen. J. Allergy Clin. Immunol..

[29] Mutschlechner S., Egger M., Briza P., Wallner M., Lackner P., Karle A., Vogt A.B., Fischer G.F., Bohle B., Ferreira F. (2010). Naturally processed T cell-activating peptides of the major birch pollen allergen. J. Allergy Clin. Immunol..

[30] Gadermaier G., Hauser M., Egger M., Ferrara R., Briza P., Santos K.S., Zennaro D., Girbl T., Zuidmeer-Jongejan L., Mari A. (2011). Sensitization prevalence, antibody cross-reactivity and immunogenic peptide profile of Api g 2, the non-specific lipid transfer protein 1 of celery. PLoS ONE.

[31] Barderas R., Villalba M., Rodriguez R. (2004). Che a 1: Recombinant expression, purification and correspondence to the natural form. Int. Arch. Allergy Immunol..

[32] Huecas S., Villalba M., Gonzalez E., Martinez-Ruiz A., Rodriguez R. (1999). Production and detailed characterization of biologically active olive pollen allergen Ole e 1 secreted by the yeast pichia pastoris. Eur. J. Biochem..

[33] Calabozo B., Diaz-Perales A., Salcedo G., Barber D., Polo F. (2003). Cloning and expression of biologically active plantago lanceolata pollen allergen Pla l 1 in the yeast pichia pastoris. Biochem. J..

[34] Rammensee H.G., Friede T., Stevanoviic S. (1995). MHC ligands and peptide motifs: First listing. Immunogenetics.

[35] Soria-Guerra R.E., Nieto-Gomez R., Govea-Alonso D.O., Rosales-Mendoza S. (2015). An overview of bioinformatics tools for epitope prediction: Implications on vaccine development. J. Biomed. Inform..

[36] Rawlings N.D., Barrett A.J., Finn R. (2016). Twenty years of the merops database of proteolytic enzymes, their substrates and inhibitors. Nucleic Acids Res..

[37] McGrath M.E., Palmer J.T., Bromme D., Somoza J.R. (1998). Crystal structure of human cathepsin S. Protein Sci..

[38] Bromme D., Bonneau P.R., Lachance P., Wiederanders B., Kirschke H., Peters C., Thomas D.Y., Storer A.C., Vernet T. (1993). Functional expression of human cathepsin S in saccharomyces cerevisiae. Purification and characterization of the recombinant enzyme. J. Biol. Chem..

[39] Lennon-Dumenil A.M., Bakker A.H., Maehr R., Fiebiger E., Overkleeft H.S., Rosemblatt M., Ploegh H.L., Lagaudriere-Gesbert C. (2002). Analysis of protease activity in live antigen-presenting cells shows regulation of the phagosomal proteolytic contents during dendritic cell activation. J. Exp. Med..

[40] Sievers F., Wilm A., Dineen D., Gibson T.J., Karplus K., Li W., Lopez R., McWilliam H., Remmert M., Soding J. (2011). Fast, scalable generation of high-quality protein multiple sequence alignments using clustal omega. Mol. Syst. Biol..

[41] Singh H., Raghava G.P. (2001). Propred: Prediction of HLA-DR binding sites. Bioinformatics.

[42] Southwood S., Sidney J., Kondo A., del Guercio M.F., Appella E., Hoffman S., Kubo R.T., Chesnut R.W., Grey H.M., Sette A. (1998). Several common HLA-DR types share largely overlapping peptide binding repertoires. J. Immunol..

[43] Kavan D., Man P. (2011). Mstools-web based application for visualization and presentation of HXMS data. Int. J. Mass Spectrom..

[44] Arnold K., Bordoli L., Kopp J., Schwede T. (2006). The swiss-model workspace: A web-based environment for protein structure homology modelling. Bioinformatics.

[45] (2017). Molecular Operating Environment (MOE).

[46] Bond C.S., Schuttelkopf A.W. (2009). Aline: A wysiwyg protein-sequence alignment editor for publication-quality alignments. Acta Crystallogr. Sect. D.

[47] Valiev M., Bylaska E.J., Govind N., Kowalski K., Straatsma T.P., van Dam H.J.J., Wang D., Nieplocha J., Apra E., Windus T.L. (2010). NWchem: A comprehensive and scalable open-source solution for large scale molecular simulations. Comp. Phys. Commun..

[48] Berman H.M., Westbrook J., Feng Z., Gilliland G., Bhat T.N., Weissig H., Shindyalov I.N., Bourne P.E. (2000). The protein data bank. Nucleic Acids Res..

